# Event-level impact of Promescent on quality of sexual experience in men with subjective premature ejaculation

**DOI:** 10.1038/ijir.2016.31

**Published:** 2016-08-25

**Authors:** K P Mark, I Kerner

**Affiliations:** 1 University of Kentucky, Lexington, KY, USA; 2 Institute for Contemporary Psychotherapy, New York, NY, USA

## Abstract

Promescent is a lidocaine-based ejaculation delaying spray that absorbs into the skin of the penis prior to sexual activity. This article aimed to evaluate the effect of Promescent on the experience of orgasm, ejaculatory latency time and quality of sexual experience (QSE). Additionally, we assessed ease of application of Promescent and the extent to which it enhanced or interrupted the sexual experience. The analytic sample consisted of 91 men with self-reported subjective premature ejaculation who were sent a sample of Promescent and completed a 14-day internet-based prospective daily electronic report. Average ejaculatory latency time was 11.16 min during product use events, compared with 6.81 min during product non-use events. Both members of the couple had an orgasm 65.6% of the time when they used the product, compared with 44.1% when they did not use the product. QSE was significantly improved on product use days (*P*<0.05). Quality also significantly improved each subsequent time the product was used (*P*<0.01). The product was reported as easy to use and did not interrupt the sexual experience. Findings suggest that the use of this topical spray significantly improves QSE and perception of partner experience, and that these improve with longer duration of use.

## Introduction

Although a number of definitions exist, premature ejaculation is characterized by three key components: timing, feeling of loss of ejaculatory control and couple distress.^1^ Premature ejaculation is often cited as the most common male sexual problem, with estimates of the prevalence ranging from 4 to 39% in the general population.^2, 3, 4, 5, 6, 7^ Waldinger and colleagues^8, 9, 10^ have proposed premature ejaculation subtypes, including lifelong premature ejaculation, acquired premature ejaculation, natural variable premature ejaculation and subjective premature ejaculation. These subtype classifications are based on intravaginal ejaculatory latency time values, the course of premature ejaculation in life and the frequency of premature ejaculation events. Both lifelong and acquired premature ejaculation present with consistent ejaculatory problems, an intravaginal ejaculatory latency time of about 1 min in lifelong premature ejaculation, about 3 min in acquired premature ejaculation and greater than about 3 min in natural variable premature ejaculation. Relevant to the current study, subjective premature ejaculation involves a cluster of inconsistent symptoms of rapid ejaculation and an intravaginal ejaculatory latency time of greater than 3–4 min. To our knowledge, there are not currently any studies focusing specifically on use of topical ejaculation-delaying agents for subjective premature ejaculation, which may be the largest proportion of men with complaints of premature ejaculation.

Potential treatments of premature ejaculation include behavioral interventions during sexual events such as stop/start, squeeze and sensate focus;^11, 12, 13^ psychological interventions that focus on sexual mindfulness, cognitive interweaves and education;^14, 15^ and pharmacological interventions such as selective serotonin reuptake inhibitors and others, which help to delay ejaculation.^16, 17, 18, 19, 20^ Promescent is a lidocaine-based topical spray that is applied to and absorbed into the penile skin prior to engaging in sexual activity with the purpose of delaying ejaculation. Similar to other lidocaine-based topical sprays, Promescent may offer a unique intervention for men who have found standard psychological and behavioral interventions to be of limited effectiveness and who are resistant to continuous daily dosing or situational dosing of selective serotonin reuptake inhibitors and due to wariness of adverse effects.

To date, researchers have not assessed the impact of Promescent as an ejaculation-delaying drug on quality of the sexual experience or published data on its efficacy in delaying ejaculation.

### Aim of the study

Utilizing a daily electronic report following each sexual encounter, this study sought to assess the impact of Promescent on the sexual experience of men who ejaculate sooner than they desire. Specifically, we aimed to assess the ease of application of Promescent and the extent to which Promescent impacted the sexual experience. Additionally, we were interested in the impact of Promescent on the experience of orgasm, ejaculatory latency time and quality of sexual experience in a sample of men who self-report premature ejaculation.

## Materials and methods

### Study design and data

Data were collected as part of a larger online study of men who self-reported that they ejaculated sooner than they wanted; 195 men completed a brief eligibility criteria survey for entrance into the daily electronic report phase of the study. As a convenience sample, these men were recruited online using social media (for example, Facebook, Twitter), email listservs and online advertisements. Of those participants, 162 men met the eligibility criteria and agreed to participate. Eligibility criteria included being a male over the age of 18, currently in a sexual relationship, living in the United States, and without any of the following medical issues: self or partner allergy to lidocaine or topical anesthetics, pregnant or possibly pregnant female partner, history of liver disease, current use of heart rhythm medication, irritated/broken skin, and/or lesions on the penis or partner's vaginal, anal, or oral tissue. Eligible participants were asked to provide their mailing address to be sent a product sample of Promescent for use during the study and instructions for participation. Once participants received the product in the mail, they were instructed to use the product every other time they engaged in sex (defined for the participant as partnered oral, anal or vaginal sex). They were also asked to report on their sexual behavior and relationship context every day for 14 days through a daily survey (a unique link was sent to them via email each day). The product sample was not blinded, participants could see the name of the product and what it was intended to do, and there was not a placebo group as part of this study design. This study design allowed for within-person comparisons in addition to between-person comparisons across the sample. Additionally, the product sample had a metered dose spray and participants were instructed to begin with three sprays applied to the glans and to titrate the dose to obtain the desired response, between 1–10 sprays per use, consistent with product insert instructions. All participants who completed four or more days of the daily electronic report were incentivized with a $20 gift card upon completion.

We chose to use a web-based data collection method because internet surveys have been shown to provide a more comfortable environment to collect data on sensitive issues such as sexual behaviors, encouraging more accurate reporting.^21^ Additionally, internet-based data collection has been shown to be methodologically equivalent to traditional data collection methods regarding validity and reliability of data, and online data collection is a more efficient way of gathering data of this nature.^22^ The study protocol was approved by the Institutional Review Board at the University of Kentucky, Lexington, KY, USA.

### Main outcome measures

#### Baseline measures

We collected information about the participants' age, sexual orientation, relationship status, education, race/ethnicity, religious affiliation, and physical and mental health status as baseline demographic measures. Additionally, relevant to the current paper, participants completed the premature ejaculation diagnostic tool, which has been demonstrated to have strong validity and reliability in assessing premature ejaculation.^23^ The premature ejaculation diagnostic tool asks participants to answer five questions on a 5-point Likert scale about difficulty delaying ejaculation (response options: ‘not difficult at all', ‘somewhat difficult', ‘moderately difficult', ‘very difficult' and ‘extremely difficult'), ejaculation before desired, ejaculation with very little stimulation (response options: ‘almost never or never (0%)', ‘less than half the time (25%)', ‘about half the time (50%)', ‘more than half the time (75%)' and ‘almost always or always (100%)'), frustration with ejaculation before desired, and perception of whether time to ejaculation impacted sexual fulfillment of partner (response options: ‘not at all', ‘slightly', ‘moderately', ‘very' and ‘extremely'). A score of 11 or more is commonly associated with the diagnosis of premature ejaculation, a score of 9 or 10 is a borderline score, and a score of 8 or less is associated with men without premature ejaculation. The Cronbach's *α* in the current sample was 0.83, indicating strong reliability. In addition to the premature ejaculation diagnostic tool, we collected information on the importance of ejaculatory control to the participant, the extent to which ejaculating too soon was bothersome for the participant, and the percentage of time during intercourse that they felt entirely in the moment.

#### Event-level measures

Participants provided data at the event level (i.e., each sexual intercourse encounter) on their sexual activity over the past 24 h. If participants did engage in partnered sexual behavior (oral sex, vaginal sex or anal sex with a partner), they were asked a series of questions about that sexual experience. Relevant to the current paper, participants indicated whether or not they used the product sample during that sexual event (‘yes' or ‘no' response options). Additionally, we collected event-level data on the following: ease of application of the product (rated on a scale of 1–10, with 1 being ‘very easy' and 10 being ‘very difficult'), the extent to which use of the product interrupted the sexual experience (‘very much', ‘somewhat', ‘a little bit' or ‘not at all'), the experience of orgasm, and participant-subjective perception of the impact of the product on their sexual experience. Finally, ejaculatory latency time was measured by the question ‘Approximately how much time (in minutes) passed between the start of penetration with your partner and ejaculation?'. We collected data on experience of orgasm and ejaculatory latency time on all days that sexual activity occurred. We only collected data about ease of product application, interruption of the experience by product use and participant perception of impact of product on sexual experience on days the participant indicated the product was used.

Participants also completed the quality of sexual experience scale^24^ on each day they engaged in sexual activity. The quality of sexual experience is a valid and reliable brief event-level measure of quality of sexual experience. Questions began with the base question of ‘Thinking about this sexual experience that you just described, would you say that it was:' and participants chose from a series of seven items scored on a 7-point semantic differential such as ‘extremely bad' to ‘extremely good' or ‘extremely unpleasurable' to ‘extremely pleasurable' or ‘extremely bad physically' to ‘extremely good physically' and so on. Scores on the quality of sexual experience (QSE) range from 7 to 49, with higher scores indicative of greater quality. The Cronbach's *α* for the QSE in the current sample was 0.93, and it has been shown to be a valid and reliable tool in prior research.^24, 25^

### Statistical analyses

Individuals who only used the product (and thus did not have any non-product use days) or individuals who never used the product (and thus did not have any product use days) were removed from the analytic data set, as no estimate of product effect could be obtained from these individuals. After those participant exclusions, the analytic sample consisted of 91 subjects. To analyze the difference between product days and non-product days, we conducted a series of random effect mixed models. Since data were collected from the same man over the course of 14 days, data points were not independent of one another in these models. Therefore, using a multilevel model approach, days were nested within individuals to account for this lack of independence of data from one day to the next within each man. Each subject received their own intercept and slope in all of the models, and all analyses were conducted using R.^26^

## Results

### Participant characteristics

A total of 91 men provided data from at least one sexual event where product was used *and* at least one sexual event where product was not used. The average age of the sample was 40.59 years (s.d.=11.16). The vast majority of the men identified as heterosexual (93.8%), with 3.1% identifying as bisexual, 2.1% as gay and 1% as questioning. While 68.8% of men described themselves as married and living with their spouse, 15.9% were partnered and living with their partner, 9.7% were partnered but not living together, 3.6% were single, 2.0% were divorced or separated. Additional participant characteristics are presented in Table 1.

### Baseline results

The average premature ejaculation diagnostic tool score at baseline was 13.48 (s.d.=4.04), indicating a clinical diagnosis of premature ejaculation to be very likely. Estimated ejaculatory latency time before product usage averaged 6.5 min in our sample. When asked about importance of ejaculatory control, 85.6% of participants indicated ejaculatory control to be extremely or very important, 11.3% felt it was moderately important and only 3.1% felt it was slightly or not at all important to them. When asked about how bothersome it was to ejaculate sooner than desired, 81% of the participants indicated it to be moderately or severely bothersome, 11.3% indicated mild to moderately bothersome, 6.2% indicated it was mildly bothersome and only 1.5% felt it was not at all bothersome. Additionally, participants indicated that during intercourse they were not entirely in the moment 73% of the time (Median=80%, s.d.=25.15) due to concerns about premature ejaculation.

### Daily electronic report results

**Figure 1:**
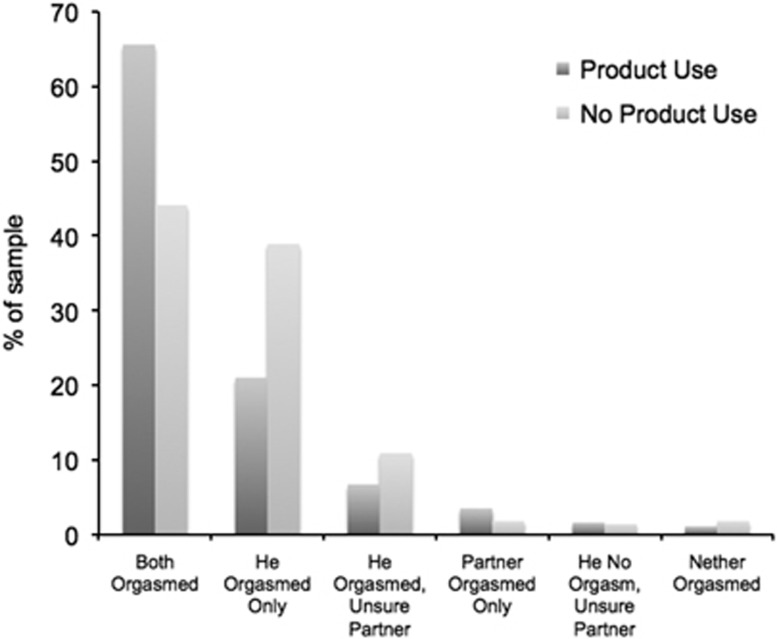
Orgasm experience with and without product use.

The average estimated ejaculatory latency time was 11.16 min during sexual events when the product was used and 6.81 min during sexual events when the product was not used; this was a statistically significant difference (*P*<0.001). When the product was used, 65.6% of sexual events resulted in both members of the couple having an orgasm, compared with 44.1% when the product was not used (*P*<0.01). Additionally, 21.0% of the men who used the product reported having an orgasm but their partner did not, compared with 38.7% on the non-product use days (*P*<0.01; see Figure 1).

On product use days, the average ease of application was 3.32 (Median=2, s.d.=3.40), indicating relative ease. The majority of participants (57.8%) indicated that the use of the product did not interrupt the sexual experience at all, with 25% indicating it interrupted a little bit, 13.7% indicating it somewhat interrupted the sexual experience, and only 3.5% indicating it very much interrupted the sexual experience. Participants largely felt the use of the product positively impacted their sexual experience, with 34.7% indicating it to be very positive and 37.4% indicating it to be a little bit positive. For 15.3% of the participants, the product did not impact their experience one way or another, and for 12.7% it negatively impacted the experience a little bit or very much.

Mixed model analyses with individuals nested within days comparing product use days to non-product use days indicated a significant impact of product use on the QSE (*P*<0.001) and scores improved as the duration of the study progressed (*P*<0.05), with the product use days giving higher QSE scores by an average of 3.80 points (see Table 2).

Next, we conducted a mixed model with the inclusion of an interaction effect between product use and the number of times the product was used. This analysis revealed that the impact of the product on QSE significantly increased by about 1.5 points, on average, each time a participant used the product (*P*<0.01). Additionally, the very first time the product was used, it significantly outperformed not using it by an average of about 2.5 points on the QSE measure (*P*<0.05; see Table 3).

## Discussion

In this examination of the impact of the ejaculation-delaying drug Promescent on sexual experience, we found that the use of the product had significant positive effects on estimated ejaculatory latency time, self and perceived partner experience of orgasm, and QSE, with the improvements increasing with the number of times the product was used. Additionally, the product was reported as relatively easy to use, with little interruption to the sexual experience in application, and a largely positive perceived impact on the sexual experience for participants. Based on this study, Promescent may be an effective solution to delay ejaculation in men with subjective premature ejaculation or for men who self-report ejaculating before they want to.

We found that not only was the QSE improved on days when the product was used, but that as men became more familiar with using the product the improvement increased. It is, however, difficult to determine causation with regard to the interaction between product use and the number of times the product was used, as it is quite plausible that subjects who performed well when using the product were far more likely to use it again as the study progressed.

Regarding orgasm, we found that product use days were more likely to elicit both members of the couple having orgasm (65.6%) than non-product use days (44.1%). To our knowledge, this is the first study to examine the experience of orgasm and perceived partner experience of orgasm related to the use of Promescent. However, it should be noted that this was the man's perception of orgasm of their partner, not the partner's report. It would be beneficial to conduct a couple-based study where the partner perception of the use of the product could be directly assessed rather than relying entirely on male perception of the partner experience. Research has indicated the detrimental impact premature ejaculation can have on relationship and sexual satisfaction and may even lead to the termination of the relationship,^27^ lending support to future research that involves couples.

In our sample, estimated ejaculatory latency time prior to product usage averaged 6.5 min. Although we did not rely on stopwatch-based intravaginal ejaculatory latency time because this was an internet-based rather than clinic-based study, the intravaginal ejaculatory latency time cutoff from the International Society for Sexual Medicine indicates that an intravaginal ejaculatory latency time of 1 min in lifelong premature ejaculation and 3 min or less in acquired premature ejaculation^28^ is significantly shorter than our sample of 6.5 min. Although the men studied had average intravaginal ejaculatory latency time well above International Society for Sexual Medicine intravaginal ejaculatory latency time criteria for lifelong and acquired premature ejaculation, these men nonetheless identified as premature ejaculators validated by premature ejaculation diagnostic tool and they met the criteria set forth by Waldinger^19, 20, 29^ as men with subjective premature ejaculation with an intravaginal ejaculatory latency time of more than 3–4 min.^29^ Additionally, research has suggested that premature ejaculation diagnostic tool is highly effective in detecting the presence of premature ejaculation and it has been supported as a valid diagnostic tool in the clinical setting.^30^ We observed that anxiety during the sexual experience was decreased with use of the spray for 40.4% of the men. While it did not impact anxiety for a roughly equal number of men (41.7%), anxiety around ejaculatory control is likely a bigger factor for men with intravaginal ejaculatory latency times of less than 1 min and with a diagnosis of lifelong or acquired premature ejaculation. However, future studies of Promescent on intravaginal ejaculatory latency time might seek to recruit a sample with intravaginal ejaculatory latency time of less than 3 min and to distinguish between men with lifelong or acquired premature ejaculation, natural variable premature ejaculation, and men with subjective premature ejaculation. Additionally, ejaculation-delaying topical drugs should be investigated in men with normal intravaginal ejaculatory latency times and premature ejaculation diagnostic tool scores as a comparison group.^29^

The study findings should be taken within the context of the methodology employed. This study design was consistent with suggestions from Waldinger^8, 9, 19, 20, 29^ that indicate subjective premature ejaculation can be studied in ways outside of the rigid design criteria required for drugs for the treatment of lifelong and acquired premature ejaculation. We did not rely on differences between baseline ejaculatory latency time and ejaculatory latency time with the use of the product. This measure, particularly when the stopwatch method is utilized, can be disruptive to the sexual experience. Since we were focused on understanding how the use of this product impacted QSE in the most natural setting possible, this measurement tool would not have been ideal. However, it would have been helpful to include a stop-watched intravaginal ejaculatory latency time measurement for the purpose of ease of comparison to prior clinical studies. Additionally, had we included a blinded control group, we could more confidently rule out a placebo effect, and this is suggested for future research. This design did allow us to examine both within and between subject assessments, where men could be compared with their own sexual experiences with and without the product use within a 14-day period. Our analytic sample size of 91 men is limited and it would be beneficial to have a larger cohort of men followed for a longer period of time in future studies. However, these 91 men completed 14 days worth of data, thus improving our statistical power considerably. Additionally, these findings should not be generalized to a clinical objective premature ejaculation sample and future research on the efficacy of this product for men with lifelong clinical premature ejaculation diagnosis. Partner experience would be a valuable addition to future studies, especially as it relates to QSE and orgasm, reduction of relational distress, transference of product from user to partner and comfort or discomfort around use and disclosure of use of product. Previous research has indicated that premature ejaculation has a significant impact on partner sexual function, satisfaction and interpersonal difficulty.^31^ Discussion that the spray was being used with the partner was not overwhelmingly common, with 57.3% of participants discussing the use of the spray with their partner and 42.7% not informing their partner on the use of the spray, and further investigation of the relational context around the use of Promescent is warranted. As premature ejaculation is a sexual problem that may require combination therapy from a biopsychosocial orientation, future studies might also seek to assess the impact of Promescent in combination with psychological, behavioral and pharmacological interventions.

## Conclusion

Promescent is an ejaculation-delaying topical agent that appears to be effective in increasing the time to ejaculation and the overall quality of the sexual experience in men with subjective premature ejaculation. Further clinical trials that include the partner perspective would be beneficial and a larger population of patients with lifelong premature ejaculation, acquired premature ejaculation, natural variable premature ejaculation, and in male volunteers with normal intravaginal ejaculatory latency times would enhance the understanding of the differential effects of this product on all men looking to delay their ejaculation.

## Figures and Tables

**Table 1:** Participant characteristics (*N*=91)

*Specification*	*Analyzed group*
Number of respondents	91
Age (years)	40.59±11.16
	
*Education*	
Grade school	0.5%
Middle school	1.0%
Some high school	2.1%
High school graduate/GED	8.7%
Some college/university	33.3%
College/university graduate	32.8%
Graduate school	19.0%
	
*Race/ethnicity*	
American Indian or Alaska Native	6.2%
Asian or Asian-American	3.6%
Black or African-American	4.6%
Native Hawaiian or Other Pacific Islander	1.0%
White or Caucasian	77.4%
Multiracial	4.6%
	
*Religion*	
Catholic	19.5%
Christian	30.8%
Hindu	1.0%
Jewish	2.6%
Mormon/Latter Day Saints	4.1%
Muslim/Islam	0.5%
Protestant	8.2%
I do not identify with any specific religion	25.6%
Other	3.6%
	
*Physical health*	
Excellent	13.3%
Very good	44.6%
Good	35.4%
Fair	6.7%
Poor	0%
	
*Mental health*	
Excellent	39.5%
Very good	40.5%
Good	16.4%
Fair	2.6%
Poor	1.0%
	
*Masturbated alone*	
Done in past week	65.1%
Done in past month	18.5%
Done in past 3 months	6.2%
Done in past year	2.6%
Done more than a year ago	2.1%
Never done with current partner	5.1%
	
*Masturbated with partner*	
Done in past week	22.6%
Done in past month	20.0%
Done in past 3 months	7.2%
Done in past year	12.8%
Done more than a year ago	12.3%
Never done with current partner	24.1%
	
*Received oral sex*	
Done in past week	46.2%
Done in past month	27.7%
Done in past 3 months	8.2%
Done in past year	6.7%
Done more than a year ago	7.2%
Never done with current partner	3.1%
	
*Gave partner oral sex*	
Done in past week	50.8%
Done in past month	24.1%
Done in past 3 months	9.2%
Done in past year	5.6%
Done more than a year ago	6.7%
Never done with current partner	2.6%
	
*Vaginal intercourse*	
Done in past week	74.9%
Done in past month	19.0%
Done in past 3 months	3.6%
Done in past year	1.0%
Done more than a year ago	0%
Never done with current partner	1.0%
	
*Anal intercourse*	
Done in past week	8.2%
Done in past month	5.6%
Done in past 3 months	7.7%
Done in past year	12.3%
Done more than a year ago	20.0%
Never done with current partner	45.1%

Note: Any percentages that do not add up to 100% represent missing data.

**Table 2:** Two mixed models predicting QSE score by product use versus non-use over time

	*Estimate*	*s.e.*	t*-value*
*Model 1*			
Intercept	39.00	1.06	36.79***
Product use	3.80	0.99	3.81***
Day	0.23	0.10	2.28*
			
*Model 2*			
Intercept	40.31	0.86	46.66***
Product use	2.45	1.05	2.34*
Product use × number of uses	1.45	0.45	3.20**

***P<0.001; **P<0.01; *P<0.05.

**Table 3:** Mixed model of product use versus non-use and number of uses on QSE score

	*Estimate*	*s.e.*	t*-value*
Intercept	40.31	0.86	46.66***
Product use	2.45	1.05	2.34*
Product use × number of uses	1.45	0.45	3.20**

***P<0.001; **P<0.01; *P<0.05.
